# Spontaneous Gender Categorization in Masking and Priming Studies: Key for Distinguishing Jane from John Doe but Not Madonna from Sinatra

**DOI:** 10.1371/journal.pone.0032377

**Published:** 2012-02-28

**Authors:** Ruth Habibi, Beena Khurana

**Affiliations:** School of Psychology, University of Sussex, Brighton, United Kingdom; Hungarian Academy of Sciences, Hungary

## Abstract

Facial recognition is key to social interaction, however with unfamiliar faces only generic information, in the form of facial stereotypes such as gender and age is available. Therefore is generic information more prominent in unfamiliar versus familiar face processing? In order to address the question we tapped into two relatively disparate stages of face processing. At the early stages of encoding, we employed perceptual masking to reveal that only perception of unfamiliar face targets is affected by the gender of the facial masks. At the semantic end; using a priming paradigm, we found that while to-be-ignored unfamiliar faces prime lexical decisions to gender congruent stereotypic words, familiar faces do not. Our findings indicate that gender is a more salient dimension in unfamiliar relative to familiar face processing, both in early perceptual stages as well as later semantic stages of person construal.

## Introduction

Faces are important social stimuli that help us identify friend from foe. In most everyday interactions, facial recognition serves as a platform upon which our interactions with the individual in question are based. Think back to the last time you unexpectedly met a friend in the street. Seeing their face permits quick recognition and access to stored knowledge about them (e.g., social status, relationship to you, common interests and so on) that is useful in guiding your behaviour toward them. Now think back to the last time you were in an unfamiliar city and looking for someone to ask directions from. Unfamiliar faces do not provide rich specific information about an individual, however they do offer plenty of useful generic information such as social category membership (e.g., gender, age, and race). This information can activate stored information about category groups allowing a best guess at the appropriate manner of interaction [1]. For example, when asking for directions, the way one chooses to address an elderly gentleman would be decidedly different than that used to address a teenage girl. Such a distinction is even more important in cultures where the language itself distinguishes between the terms chosen to address an individual as a function of their gender, or their age relative to the addresser (for example, in many languages (e.g., Hindi) the choice of verb and/or pronoun changes as a function of the gender of individual being addressed or their age relative to the addresser). As social categories are the most useful information we can get from unfamiliar faces, we propose that category information is more salient and therefore prioritized in terms of processing. For familiar faces on the other hand, processing resources may be directed towards recognition as this provides far more nuanced information for guiding social interactions. Indeed recognition has been proposed to occur via a specialised face recognition processing route comprised of face recognition units (FRUs) and person identity nodes (PINs) [2], [3], which is activated after even the briefest of exposure to a familiar face [4], [5]. According to the IAC model [3], activated PINs trigger related semantic information units (SIUs) that store personal information about the face. This entire face recognition process is proposed to occur separately to the processing of other information about the face such as expression or indeed social category information such as gender. Therefore if processing via this route takes priority on seeing a familiar face, then it is reasonable to assume gender categorisation would be less prominent or doesn't remain ‘online’ because of competing semantic information. However, support for dual route models is not unanimous and some argue that in fact all information from a face is processed via a single route [6], [7]. In this case whether or not a face is familiar, gender categorisation would be the same but truncated for unfamiliar faces as specific information doesn't exist for them.

Face gender can be explicitly categorised within a few hundred milliseconds of seeing a face [8]. But in order to measure if gender is still processed when it is not directly relevant to the task, more subtle methods need to be employed. One indirect method suited to investigating the incidental processing of gender is perceptual masking, as the interference caused by a subsequent mask provides a measure of the nature of initial coding of a face [9]. A split second view (as little as 27 ms) of a face presented without a mask is sufficient to extract the information needed to distinguish it from other faces [10]. However, when followed immediately by another stimulus, performance on both discrimination and recognition tasks can be severely impaired [9], [11], [12], [13], [14], [15].

Importantly, in the present context, the extent to which a new stimulus masks the initial presentation of a face depends on the visual nature of the face and the masking stimulus. Noise masks and other non-face objects only minimally hinder processing [13], [14], whereas masks made of whole upright faces cause significant impairment in the successful processing of target faces [9], [11], [12], [13], [14]. Intermediate levels of interference are observed with masks comprised of face parts, upside-down faces and faces with scrambled features [9], [12], [13]. Such findings have been accounted for in terms of the ease with which masks can be distinguished from targets: Masks that can easily be categorized as new and distinct objects render reduced interference [13], [16]. Therefore, we used the following logic in the present experiments: The degree of impairment caused by masks of varying similarity to the target can be used as a measure of the facial dimensions that are encoded early, as only dimensions that have been processed prior to the onset of the mask can be used to distinguish the target from the mask. For example, ¾ profile faces are masked more completely by similarly oriented face masks relative to those oriented in the opposite direction suggesting an early coding of facial orientation [11]. Furthermore, it appears that the level of masking depends not simply on the physical similarity between target and mask, but also on the importance of the dimensions in which the target and mask are similar [12], [17]. For instance, the configuration of features is known to be key to the processing of upright faces. As such, processing of upright target faces is disrupted more when the mask is a whole upright face than when it is made up of scrambled facial features. However upside-down faces are thought not to engage configural processes. Accordingly, there is no difference in the masking of upside-down faces whether masks are whole or scrambled upside-down faces [12]. Therefore, if gender is a key dimension in the representation of unfamiliar face processing, then for unfamiliar face targets oppositely gendered face masks should be easier to distinguish and thereby cause less masking than same gendered face masks. For familiar faces, on the other hand, if gender is not an important dimension in the representation of the face, there should be no difference between masking by faces of the same or opposite gender to that of the target face. These predictions were tested in Experiment 1.

If gender categorisation is indeed less salient in the initial processing of familiar faces, then one can also make a prediction about gender stereotypes: Gender stereotypes should be less activated on seeing a familiar face than on seeing an unfamiliar face. Previous research has shown that unfamiliar faces spontaneously activate gender stereotypes [18], [19]. For example, in one study participants were presented with a lexical decision task in which words were either male or female stereotyped words (e.g., “strong” or “pink”). Before each word a briefly presented unfamiliar face appeared that participants were instructed to ignore. Despite the instruction to ignore the face, it's gender was encoded and activated related stereotypes such that congruently stereotyped words were primed [18]. Thus, gender categorisation is highly important in evaluating unfamiliar faces. Based on the reasoning presented above, in Experiment 2 we tested the prediction that familiar faces would not elicit similar stereotype priming.

## Methods

### Ethics Statement

The study was examined and passed by the University of Sussex, School of Life Sciences Ethics Committee. Participants (82 in total: 12 male; mean age 22 years) were recruited from the University of Sussex Psychology participant pool and were given course credits for taking part. All provided written informed consent prior to taking part.

### Experiment 1: Masking experiments

**Figure 1:**
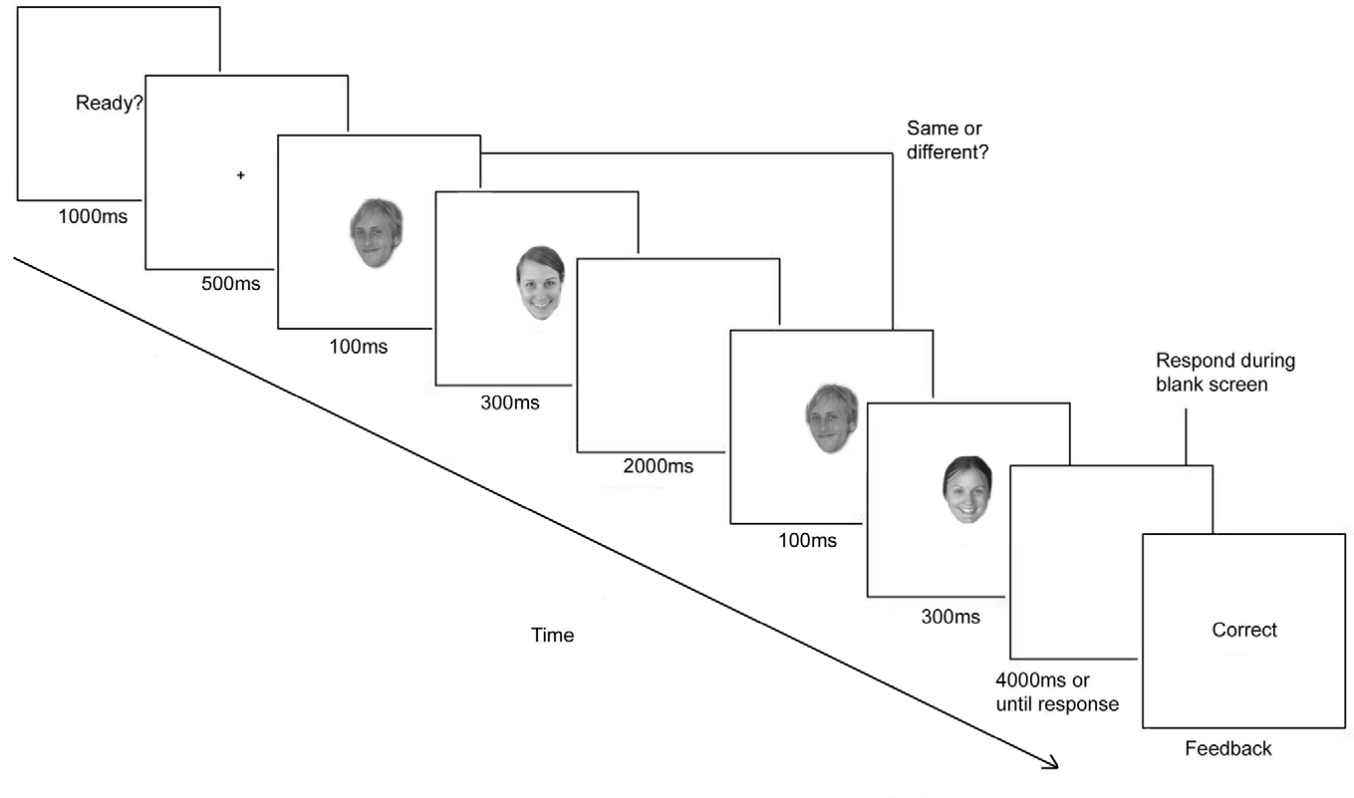
Experiment 1 trial sequence. Participants were required to decide if the two targets were the same face or not.

Figure 1 shows the sequence of events in a trial from Experiment 1. Stimuli consisted of greyscale images of 64 unfamiliar faces and 64 familiar faces (all Caucasian). In Experiment 1a all faces (targets and masks) were unfamiliar; in 1b all faces were familiar. In Experiment 1c familiar target faces were masked by unfamiliar faces and vice versa. Unfamiliar faces were taken from an online database of unknown model and actor headshots in order to try and match the quality and attractiveness of the familiar faces. Familiar faces consisted of well known celebrity faces, such as Brad Pitt and Britney Spears. All faces were either smiling or wore a neutral expression and there was a similar mix of expressions in the familiar and unfamiliar, and male and female faces. To ensure the faces were familiar to each participant, at the end of the experiment each participant was asked to identify each face either by name or an identifying piece of information (such as a film they had been in). All participants recognised at least 90% (58/64) of the familiar faces; mean recognition rate was 97%. The entire list of celebrities used is available in the additional material (see Figure S1). Image backgrounds were removed and the hair of all the faces was cropped such that all faces (male and female) had short hair rendering them not easily distinguishable based solely on their silhouettes.

In each trial, a briefly presented (100 ms) target face was immediately masked by the presentation of a second face (300 ms) and then after a blank screen interval (2000 ms) it was followed by a second target (100 ms) and mask (300 ms). Participants had to indicate if the two target faces were the same or different. Targets within a trial were always of the same gender. On half the trials, the mask faces (which were always of the same gender) were the same gender as the target faces (gender match condition) and on the other half, they were the opposite gender (gender mismatch condition). Feedback was given at the end of each trial in the form of a screen saying ‘Correct’ or ‘Incorrect’. Participants completed 12 practice trials before the main experiment consisting of 144 trials. Each individual face appeared 9 times in the experiment − 4/5 times as a target and 4/5 times as a distracter. The order and combination of faces was randomised for each participant.

### Experiment 2: Stereotype priming

**Figure 2:**
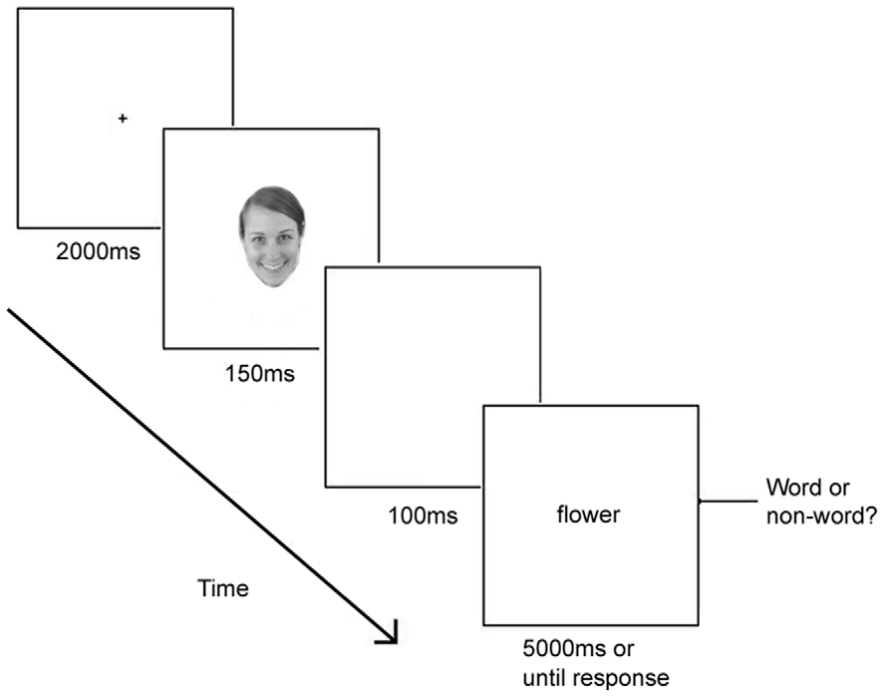
Experiment 2 trial sequence. Participants were required to decide if the letter string was a real word or not and were told to ignore anything else that appeared on the screen.

In this experiment, the task was to make a lexical decision [18] in which all the words were gender stereotyped (e.g., “pink” or “strong”). The words, 12 stereotypically female and 12 stereotypically male, were taken from Blair & Banaji [20], and the non-word letter strings were the words rearranged to make pronounceable non-words. Each word was presented in the centre of the screen for 5 seconds or until the participant responded. Before each letter string, a briefly presented (150 ms) familiar or unfamiliar face appeared on the screen, which participants were instructed was irrelevant to the task. The face stimuli consisted of greyscale images of 12 familiar (6 male) and 12 unfamiliar (6 male) faces. Each face appeared once with a gender congruent word, once with a gender incongruent word and once with a non-word. The face-word/non-word pairs were randomised for each participant. Figure 2 shows a typical trial.

## Results

### Experiment 1: Masking experiments

#### Experiment 1a

Given previous findings of the gender of unfamiliar faces being automatically encoded [18], [21], we reasoned that masking of unfamiliar face targets should be greater from same gendered face masks than opposite gendered face masks. 16 participants (6 male) completed the masked face matching experiment described above, with unfamiliar target and mask faces for course credits.

#### Results & Discussion

To measure the amount of interference caused by the masks, mean accuracy (d′) scores were calculated and entered into a 2×2×2 mixed ANOVA (participant gender × target gender × mask gender). As predicted there was a significant interaction between target gender and mask gender, F (1, 14) = 6.42, p = 0.02, in that accuracy was greater when the mask was of the opposite gender to the target. The only other significant effect was greater accuracy for male targets (d′ = 2.9) compared to female targets (d′ = 2.7), F (1, 14) = 5.41, p = 0.04. However as this difference did not affect the target by mask interaction, target and mask genders were collapsed thus leaving two conditions (gender match and gender mismatch). Likewise as there was no effect of participant gender it was not considered further. When these two conditions were entered into a repeated measures t-test, accuracy was significantly higher when face targets were of opposite gender to the face masks, *t* (15) = −2.97, p = 0.01. These findings support the view that encoding of facial gender is an obligatory process, as it occurs even when gender is not directly relevant to the task at hand [6], [7].

The gender of an unfamiliar face is a key distinguishing feature that serves as a vital input for social interactions. Since familiar faces can be recognised, the relative salience of dimensions such as gender may be less. For example, if your friend Katie (say) is a football fan, then by recognising her, the more normative classification of Katie as a female, and associated stereotypes, may be bypassed to engage in conversation about her favourite team. If gender is not a salient dimension of familiar faces, then it may have less of an effect on the masking of familiar face targets. This was investigated in Experiment 1b.

#### Experiment 1b

16 new participants (1 male) completed the same procedure as that in Experiment 1a, with the exception that all face targets and masks were familiar.

#### Results & Discussion

**Figure 3:**
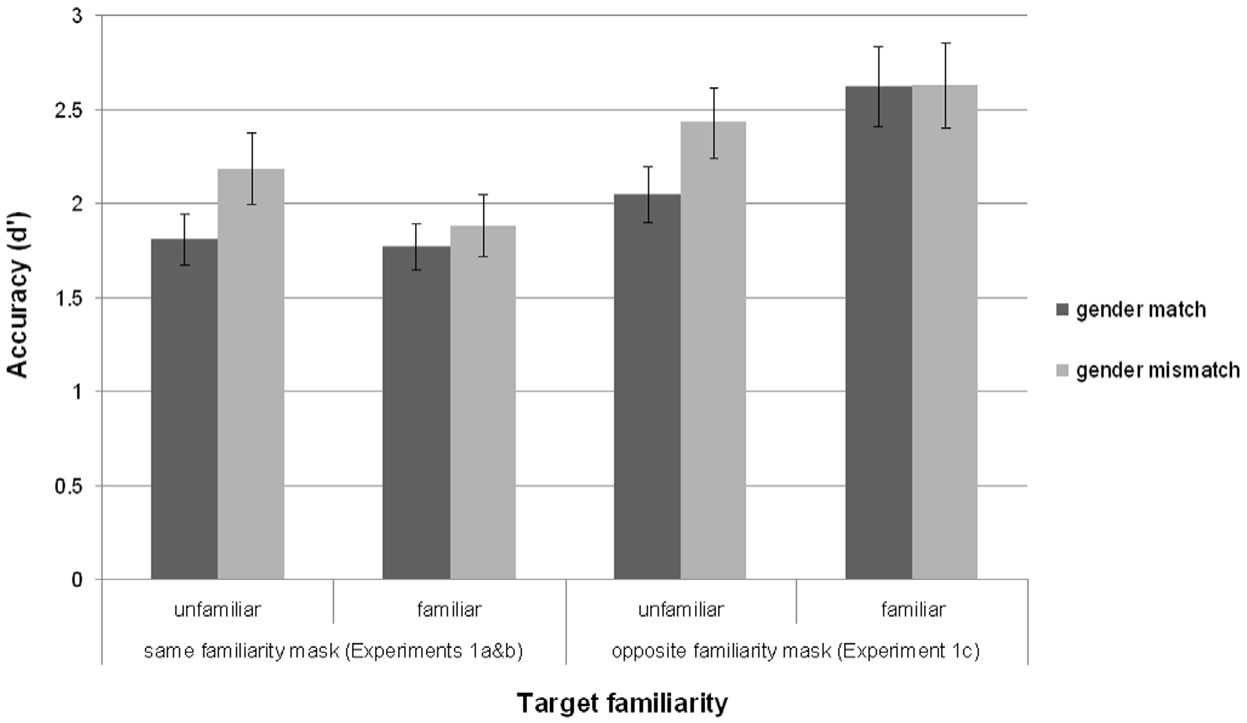
Experiment 1 accuracy scores. Face masks were of the same (gender match) or the opposite (gender mismatch) gender to the face targets. Targets and masks were of the same familiarity in Experiments 1 a & b and of opposite familiarity in Experiment 1 c. Error bars indicate standard error of the mean.

Accuracy (d′) scores (Figure 3) showed no significant difference between gender match and mismatch conditions, *t* (15) = −0.98, p = 0.34. Although not significant there is still a trend in the same direction as for the unfamiliar faces in Experiment 1a. When the data from Experiment 1a and b were combined in a mixed ANOVA the overall significant effect gender matching, F (1, 30) = 7.02, p = 0.01, was not qualified by a gender match × familiarity interaction, F (1, 30) = 1.21, n.s. Thus, although it appears the gender of familiar faces is less pronounced in the initial encoding of the face, due to the smaller masking effect on familiar faces, these results do not rule out gender processing of familiar faces. However caution should be exercised given that there were two independent participant groups.

The fact that familiar target faces were masked by familiar faces could warrant an entirely different explanation. Unlike unfamiliar faces, familiar faces can be personally identified and hence trigger semantic knowledge, which could have engaged the participants' attention. As the face masks were exposed for three times longer than the target faces, there exists a greater likelihood of them being identified. Therefore, it is possible that even if the gender of the familiar targets was coded early as with unfamiliar faces, the familiar masks were simply more distracting. This could impede participants' ability to maintain the representation of the target regardless of whether gender had been used to distinguish the mask as a new individual. Consequently, the greater ability of familiar masks to vie for attentional resources may have caused the smaller difference in performance in the gender match and mismatch conditions for familiar faces. Indeed this could also account for the somewhat counterintuitive overall lower accuracy in matching of familiar targets relative to unfamiliar targets (as seen in Figure 3). This possibility was addressed in Experiment 1c.

#### Experiment 1c

In order to test if familiar face masks had a particularly detrimental effect on target matching performance, both familiar and unfamiliar face targets were presented with face masks of the opposite familiarity (i.e., familiar targets had unfamiliar masks and vice versa). If the lower accuracy and lack of effect of gender of the face masks for familiar targets was due to familiar face masks being too distracting, then the pattern of results seen in Experiment 1a & b should be reversed. Specifically, overall accuracy should be lower with unfamiliar targets and the gender effect seen in Experiment 1a (i.e., lower accuracy when target and mask were the same gender) should no longer be observed. On the other hand, accuracy for familiar targets should increase compared to Experiment 1b and the effect of the gender of the face masks should become apparent. 16 new participants (2 male) completed two blocks, one with familiar targets and unfamiliar masks and the other with unfamiliar targets and familiar masks. The order of these blocks was counterbalanced across participants.

#### Results & Discussion

A 2×2 repeated measures ANOVA showed significantly higher accuracy (d′) scores for familiar than unfamiliar targets *F* (1, 15) = 9.38, p = 0.008, and a significant interaction between familiarity and gender-matching *F* (1, 15) = 5.82, p = 0.03. As can be seen in Figure 3, accuracy for familiar targets did increase with unfamiliar masks (Mean d′ = 2.6 compared to Mean d′ = 1.8 in Experiment 1b) but did not result in a significant difference in the masking effect between masks of the same versus the opposite gender, *t* (15) = 0.04, p = 0.97, replicating the null result from Experiment 1b. Conversely, familiar masks did not lower the overall accuracy of unfamiliar targets (Mean d′ = 2.2 vs. Mean d′ = 2.0), and a gender of mask effect was still observed, *t* (15) = 3.15, p = 0.007, replicating the result of Experiment 1a. Therefore, it is evident that the lack of differential gender masking with familiar face targets cannot be due to the greater potential of familiar face masks to distract.

We come back then, to our initial proposal that gender is more important in the processing of unfamiliar compared to familiar faces. This makes good sense when considering inputs to social interactions. For unfamiliar faces that cannot be individually identified, dimensions such as gender are necessary for building up a representation of who the person is and how to engage with them. Gender and other such dimensions will therefore need to be coded early on to enable the viewer to access stored semantic information about the social groups that can inform interactions [1]. Hence gender of unfamiliar faces is quickly available to distinguish the mask from the target. However familiar faces that can be individually recognized, rely less on gender as a distinguishing dimension. For familiar faces, identification is more informative than whether the face is male or female, so recognition is prioritised over gender categorisation.

### Experiment 2: Stereotype priming

If, as Experiment 1 suggests, gender categorisation is less of a priority on seeing familiar faces then gender stereotypes may not be spontaneously activated by familiar faces. Thus the previously observed stereotype priming by unfamiliar faces [18] should not be observed for familiar faces.

#### Results & Discussion

**Figure 4:**
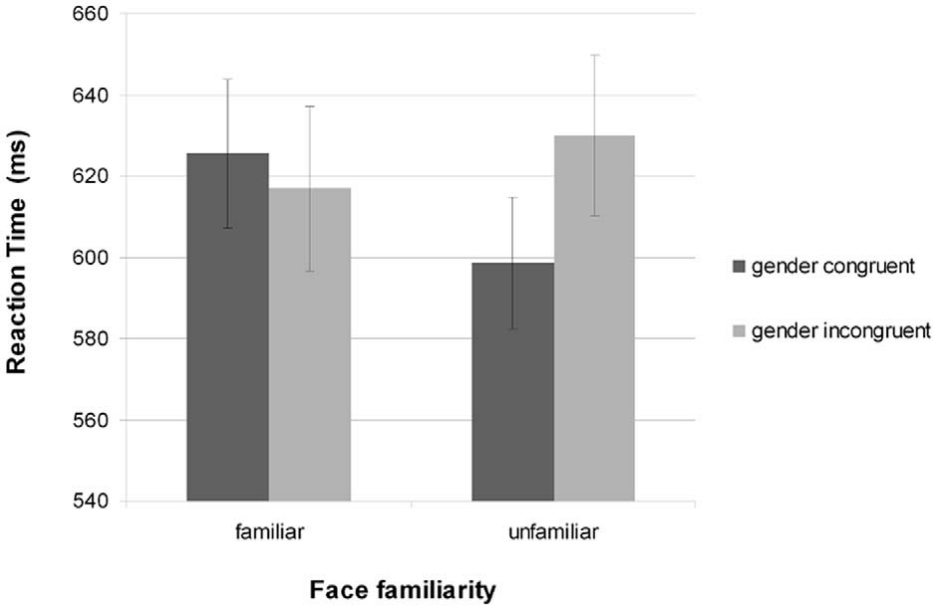
Experiment 2 average reaction times. Participants performed a lexical decision task in which real words were gender stereotyped. All words were preceded by familiar and unfamiliar faces of congruent or incongruent gender. Median reaction time scores were calculated and bars represent means of participants median RTs. Error bars indicate standard error of the mean.

Median reaction times were calculated for responses to gender stereotyped words following congruently and incongruently gendered familiar and unfamiliar faces. Errors were low (3.2%) and showed no systematic differences across conditions. Error trials were excluded from the reaction time analysis and not considered further. A 2×2 (word-face gender congruency × face familiarity) repeated measures ANOVA revealed no significant main effects but did show a significant interaction between conditions, *F* (1, 33) = 5.13, p = 0.03. This interaction was based on significant stereotype priming from unfamiliar faces, *t* (33) = 2.64, p = 0.01, but not from familiar faces, *t* (33) = −0.75, p = 0.46, as can be seen in Figure 4.

The results from Experiment 2 replicated previous findings that gender stereotyped words were classified as words faster when preceded by an unfamiliar face of the congruent gender than when preceded by an unfamiliar face of the incongruent gender [18]. However, when the unattended faces were familiar there was no difference in reaction time to gender congruent or incongruent words. These priming results corroborate the findings of Experiment 1 and lend further support to the position that while gender is an important dimension of the initial representation built up on seeing an unfamiliar face; this is not the case for familiar faces.

## Discussion

Overall the results from both the masking and the priming experiments support a clear distinction between familiar and unfamiliar face processing. In Experiment 1, unfamiliar target faces were masked more by faces of the same gender, suggesting that gender is an important dimension in the early representation of unfamiliar faces. Experiment 2 revealed that the early coding of unfamiliar face gender significantly primes responses to congruently gender stereotyped words. We argue that the rapid coding of gender activates relevant stereotypes in the service of guiding social interactions. On the other hand, faces of the same and opposite gender equally masked familiar target faces. Therefore, it seems that gender has a less important role in the encoding of familiar faces. In line with this, familiar faces did not prime congruently gender stereotyped words, reinforcing the position that the gender of familiar faces is not key in ascertaining how to interact with a familiar person.

The findings from Experiment 1 extend the previous findings of face masking studies [9], [11], [12], [14] by demonstrating that even when face masks are normally configured faces, there are still differences in the masking of the target depending on the similarity between target and mask in terms of face category (i.e., gender). It could be argued that the greater masking of unfamiliar target faces by faces of the same gender found in Experiments 1a & c was simply due to same gender faces being more physically alike than opposite gender faces. However for this to be true, the same results should have been found with familiar target faces (Experiments 1b & c). As this was not the case, our findings extend previous ones showing that in face processing, similarity along conceptually important dimensions, rather than purely physical similarity between target and mask, is key [12], [17]. The idea is further supported when the results for familiar targets in Experiments 1b and c are considered together. Here it appears that the conceptually important dimension of familiarity (i.e., the first step in recognition) was key, with accuracy being lower when the distracters matched the targets on this dimension.

The fact that accuracy on the matching task was lower when familiar targets were masked by familiar masks than unfamiliar masks can be readily explained by the IAC model of face recognition [3]. This model can even account for the somewhat surprising finding in Experiment 1b of lower accuracy of matching familiar compared to unfamiliar faces (in Experiments 1a and c). According to the model, activation of a FRU leads not only to the activation of the corresponding PIN and SIUs, but also to suppression of all other FRUs (and consequently their related PINs and SIUs). Thus, when a familiar mask appears the activation of its FRU suppresses the FRU of the target face making the matching task harder. As unfamiliar faces have no FRUs, unfamiliar targets are not affected by the familiarity of the target. Likewise unfamiliar masks cannot suppress the FRUs of familiar targets, hence the much greater accuracy for familiar target matching in Experiment 1c. Nonetheless, even with this familiarity effect accounted for there was still no gender effect for familiar targets and so it is clear that gender plays a less central role in the perception of familiar faces.

Although we have argued our findings suggest that gender is a less important dimension in the processing of familiar faces, they cannot conclusively rule out the possibility of early coding of gender of familiar faces. It is indeed possible that the gender of familiar faces is encoded just as early as for unfamiliar faces but the recognition of the face overwrites the effects of gender categorisation. One way to test this could be to present the faces for a shorter amount of time to try to tap into the gender categorisation before it is set aside. However, it has previously been found that the face recognition route is automatically activated on seeing a familiar face, with FRUs being activated in as little as 17 ms [2], [3], [4], [5]. Therefore it follows that the gender of the mask face couldn't have an effect on the level of masking with familiar faces. Once the FRU associated with the target face is activated, no distracter – male or female – would match it, rendering gender irrelevant. Even if gender is encoded it evidently plays a less important role in the early representation of familiar as compared to unfamiliar faces. This becomes especially clear when considering the results of Experiment 2. Here we found unequivocal evidence that the gender of unfamiliar faces is not only encoded but also activates related stereotypes in the semantic knowledge pool. These stereotypes were then able to prime the categorisation of congruently gender stereotyped words. For familiar faces no such priming was observed, suggesting that even if the gender of familiar faces is encoded, it is not prioritised in the representation of the face, and so related stereotypes are not activated. But in the same way that gender categorisation leads to activation of stereotypes, the identification of familiar faces would lead to the activation of stored semantic information about the person via the PINs and SIUs [2], [3]. This could provide priming for words associated with the semantic information so that even if gender stereotypes were activated, the gender priming is disguised. For example the stereotypically male word “strong” may be primed more so by a picture of Arnold Schwarzenegger's face, where the semantic knowledge would also facilitate the processing of this word, than a picture of Michael Jackson's face even though they are both male faces.

Although reducing presentation times of faces may not clarify whether the gender of familiar faces is in fact categorised early on in processing, closer consideration of the time course of processing might still be the best way forward. A clearer picture of gender categorisation in familiar faces could perhaps be gained from looking at event-related potentials (ERPs). These have the benefit of being able to directly measure processing of dimensions not relevant to the task at hand and being particularly sensitive to the time course of processing. Therefore, dimensions that may be disguised when using behavioural measures can become apparent. If gender is processed in familiar faces just as in unfamiliar faces, gender related ERPs should be similarly activated for familiar and unfamiliar faces. Several ERP studies have already suggested that gender categorisation in unfamiliar faces occurs independently of the focus of attention [22], [23], [24], [25] but as yet there are no studies that specifically look at the incidental processing of gender in familiar faces. While this is beyond the scope of this paper, it is an interesting direction for future research.

The experiments in this paper offer new insights into differences in early processing of familiar and unfamiliar faces. For unfamiliar faces, the gender of the face is an important dimension that is coded early, making it a useful distinguishing feature. Furthermore, this coding leads to the activation of gender stereotypes resulting in the priming of congruently gender stereotyped words. The present findings do not completely rule out the possibility of early processing of gender in familiar faces, however they do indicate gender is less important than in the processing of unfamiliar faces. We argue that this is due to more detailed information about the individual being available through recognition thereby reducing the reliance on social category stereotypes to guide interaction. Previous studies of face masking have provided support for an early distinction between faces and other objects and the importance of configuration in initial representations of faces. Our results highlight an additional early distinction between familiar and unfamiliar faces: Gender is key in distinguishing between Jane and John Doe, but not between Madonna and Sinatra. It would be interesting to explore whether other facial categories such as age and race behave similarly. Furthermore, these findings endorse and elucidate previous findings on how experience and individuation results in a reduced other-race deficit in recognition [26] and reduced racial bias [27]. We expand on these by providing evidence for gender stereotypic thinking being muted, if not absent for known individuals. In other words, prejudicial inferences can be minimized by getting to know someone.

## Supporting Information

Figure S1 **List of celebrities used as familiar faces.**
(DOC)
