# Supplementary material for: Spontaneous Gender Categorization in Masking and Priming Studies: Key for Distinguishing Jane from John Doe but Not Madonna from Sinatra
Source: PLoS One. 2012 Feb 28;7(2):e32377. doi: 10.1371/journal.pone.0032377 (PMC3289646; doi:10.1371/journal.pone.0032377)
Supplement: Figure S1 — List of celebrities used as familiar faces. (DOC) [file pone.0032377.s001.doc]

List of celebrities used as familiar faces

| Angelina Jolie | Adam Sandler |
| --- | --- |
| Britney Spears | Anthony Hopkins |
| Cate Blanchett | Arnold Schwarzenegger |
| Catherine Zeta Jones | Ben Stiller |
| Emma Bunton | Bill Clinton |
| Gwyneth Paltrow | Brad Pitt |
| Jennifer Aniston | Bruce Willis |
| Judi Dench | David Beckham |
| Kate Winslet | George W. Bush |
| Keira Knightley | Jack Nicholson |
| Julia Roberts | Jim Carey |
| Cameron Diaz | John Travolta |
| Nicole Kidman | Johnny Depp |
| Sarah Jessica Parker | Matt Damon |
| Victoria Beckham | Nicholas Cage |
| Christina Ricci | Robbie Williams |
| Kirsten Dunst | Robert DeNiro |
| Michelle Pfeifer | Tom Cruise |
| Scarlet Johansson | Russell Crowe |
| Sandra Bullock | George Clooney |
| Katie Holmes | Leonardo DiCaprio |
| Jennifer Lopez | Justin Timberlake |
| Hilary Clinton | Hugh Grant |
| Drew Barrymore | Ewan McGregor |
| Princess Diana | Elvis Presley |
| Demi Moore | Colin Farrell |
| Courtney Cox | Colin Firth |
| Claire Daines | Clive Owen |
| Calista Flockhart | Christopher Walken |
| Audrey Hepburn | Bill Murray |
| Andi MacDowell | Ben Affleck |
| Alison Hannigan | Antonio Banderas |
